# Acute Bladder Necrosis after Pelvic Arterial Embolization for Pelvic Trauma: Lessons Learned from Two Cases of Immediate Postembolization Bladder Necrosis

**DOI:** 10.1155/2016/7594192

**Published:** 2016-09-05

**Authors:** Samuel Washington, E. Charles Osterberg, Sean P. Elliott, Adam B. Hittelman, Benjamin N. Breyer

**Affiliations:** ^1^Department of Urology, University of California San Francisco, San Francisco, CA, USA; ^2^Department of Urology, University of Minnesota, Minneapolis, MN, USA; ^3^Department of Urology, Yale University, New Haven, CT, USA

## Abstract

We report two cases of acute bladder injury with bladder neck necrosis identified during the initial operative evaluation and within the early postprocedural period in patients with significant pelvic trauma requiring pelvic vascular embolization. To our knowledge, this is the first report of bladder neck necrosis found during the initial intraoperative surgical evaluation or early postoperative setting.

## 1. Introduction

After significant pelvic trauma, multiple interventions aimed at obtaining vascular control can be utilized including electrocauterization, ligation of bleeding vessels, pelvic packing, and, in select cases, embolization of bleeding vessels [1–3]. Embolization of pelvic arteries continues to be the mainstay of treatment for uncontrolled hemorrhage secondary to pelvic bleeding in critically ill patients. Previous studies have reported the efficacy and safety of pelvic artery embolization [3, 4], with known potential complications such as impotence, gluteal muscle necrosis, and bladder necrosis [5]. Bladder necrosis is a result of embolization of the internal iliac or hypogastric arteries, unilateral or bilateral, with necrosis typically identified several weeks after the initial injury. Various cases of subacute bladder necrosis after embolization have been reported in the literature, typically four weeks after embolization [2, 6]. We report two cases of acute bladder injury with bladder neck necrosis identified during the initial operative evaluation and within the early postprocedural period in patients with significant pelvic trauma requiring pelvic vascular embolization. To our knowledge, this is the first report of bladder neck necrosis found either during the initial intraoperative surgical evaluation or during the early postoperative setting.

## 2. Case  1

**Figure 1:**
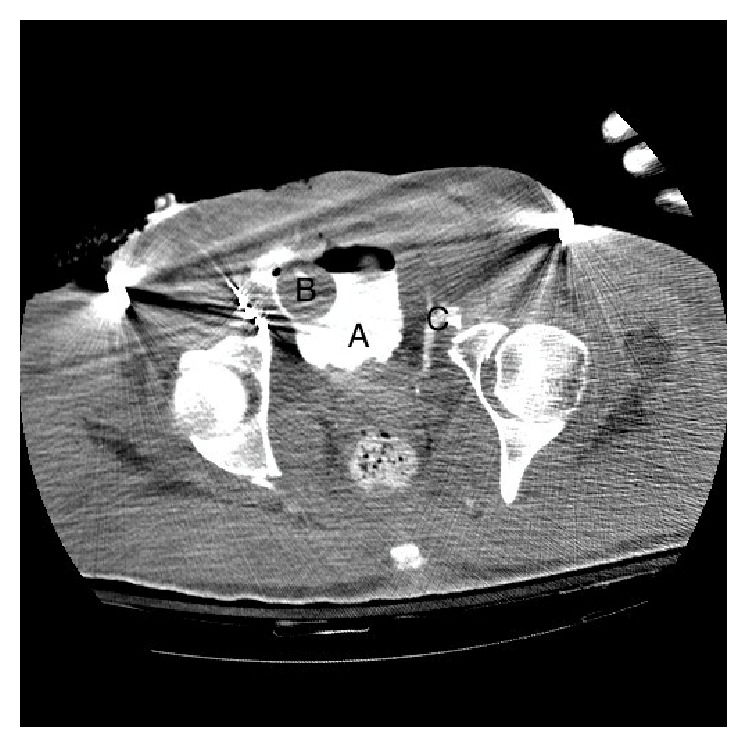
CT A/P demonstrating extent of injuries noted on presentation. A: bladder with contrast. B: balloon of suprapubic tube. C: contrast extravasation outside of the bladder.

A 53-year-old African American man involved in a pedestrian versus automobile accident presented to the emergency room. He was alert and responsive with persistent hypotension requiring multiple transfusions of blood products. He underwent a computed tomography (CT) scan with trauma protocol demonstrating multiple injuries including bilateral pneumothoraces, bilateral Grade III-IV renal lacerations, a severe pelvic fracture with disruption of the pubic symphysis, and a urethral and pelvic hematoma. Active extravasation of contrast was noted from the bilateral internal iliac arteries. The patient was taken emergently to Interventional Radiology (IR). Pelvic angiography revealed transection of the left internal iliac artery with ongoing extravasation of contrast from the right internal iliac artery. He underwent selective embolization of bilateral internal iliac arteries, right inferior epigastric artery, and the left L4 lumbar artery. Despite this intervention, he was noted to have continual pelvic bleeding. A suprapubic tube was placed and positioned well with confirmation by flushing the catheter though it was noted to have little drainage after placement. CT cystogram demonstrated the significant pelvic trauma, including extravasation of contrast, confirming intraperitoneal bladder rupture (Figure 1). The bilateral renal lacerations were managed conservatively with close observation. He was taken to the operating room later that day with a concern for abdominal compartment syndrome. An exploratory laparotomy was made and a significant amount of blood and clot was evacuated from around the bladder. A prominent pelvic hematoma was noted in the inferior portion of the space of Retzius. An anterior cystotomy was performed on the patient to rule out a possible bladder injury. A small extra peritoneal bladder rupture was identified and repaired primarily with a running 3-0 vicryl suture. Attempted Foley catheter placement was unsuccessful and a posterior urethral disruption was identified under direct visualization via cystoscopy. The bladder was closed in two layers with a suprapubic tube left in place. The abdomen was left open and packed by the trauma service and the orthopedic service stabilized his pelvis with external fixation. In total, he had received 62 units of packed red cells, 41 units of fresh frozen plasma, 10 six-packs of platelets, and several doses of Factor VII during resuscitation.

He was monitored in the Intensive Care Unit (ICU) for two days and then taken back to the operating room for washout, removal of packing, and attempted closure of the abdomen. There was no evidence of ongoing bleeding. The suprapubic tube was noted to be in place. Additional surgical drains were placed and closure was successful. A wound vacuum was placed over the abdomen.

**Figure 2:**
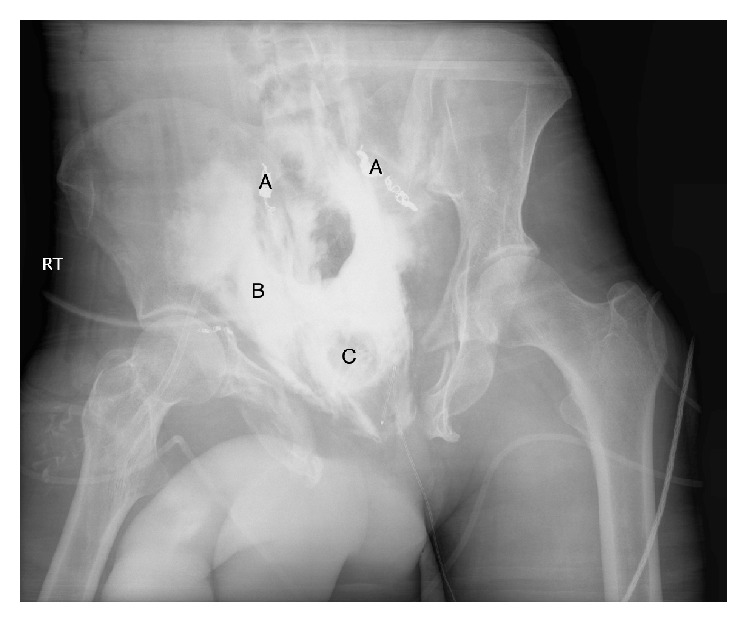
Cystogram demonstrating extravasation and contrast and urine out of the bladder. A: embolization coils in bilateral internal iliac arteries. B: extravasation of contrast outside of the bladder. C: suprapubic tube balloon.

**Figure 3:**
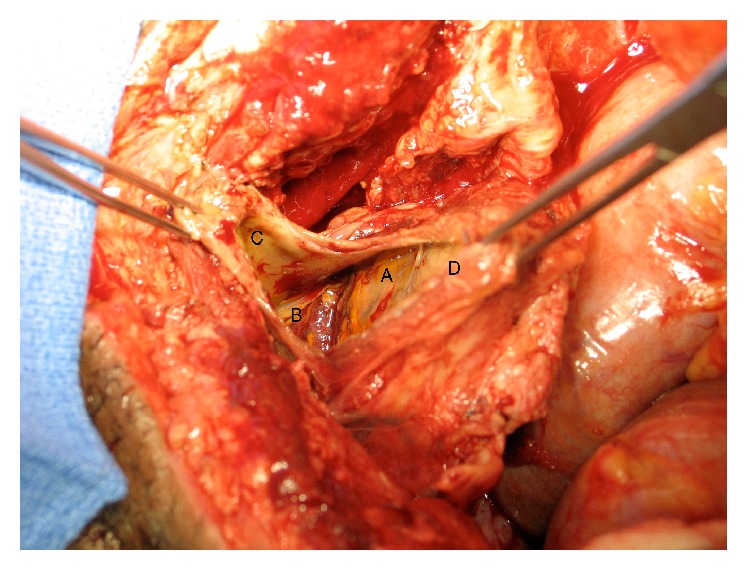
Intraoperative photo of bladder injury. A: necrotic tissue. B: trigone. C: anterior bladder wall. D: posterior bladder wall.

He continued to require ICU level care and was noted to have significant urine leakage through the pelvic drains. A cystogram 48 hours after the initial injury demonstrated a leak from the bladder and elevated fluid creatinine from the surgical drains confirmed the diagnosis of a urine leak (Figure 2). A follow-up CT scan one week after his admission demonstrated improvement of the bilateral renal lacerations, without extravasation of contrast on either side. He was taken back to the operating room on hospital day 12 from his initial presentation for pelvic and bladder exploration. Intraoperatively, he was noted to have necrosis of the bladder with sparing of the trigone and prostate (Figure 3). Exposed pelvic bone was visualized with surrounding necrotic pelvic floor musculature. A simple cystectomy was performed and the bilateral ureters were ligated at the bifurcation of the iliac arteries. IR placed bilateral nephrostomy tubes via a percutaneous approach. He was soon discharged and returned for ileal conduit urinary diversion 7 months after his initial injury.

## 3. Case  2

**Figure 4:**
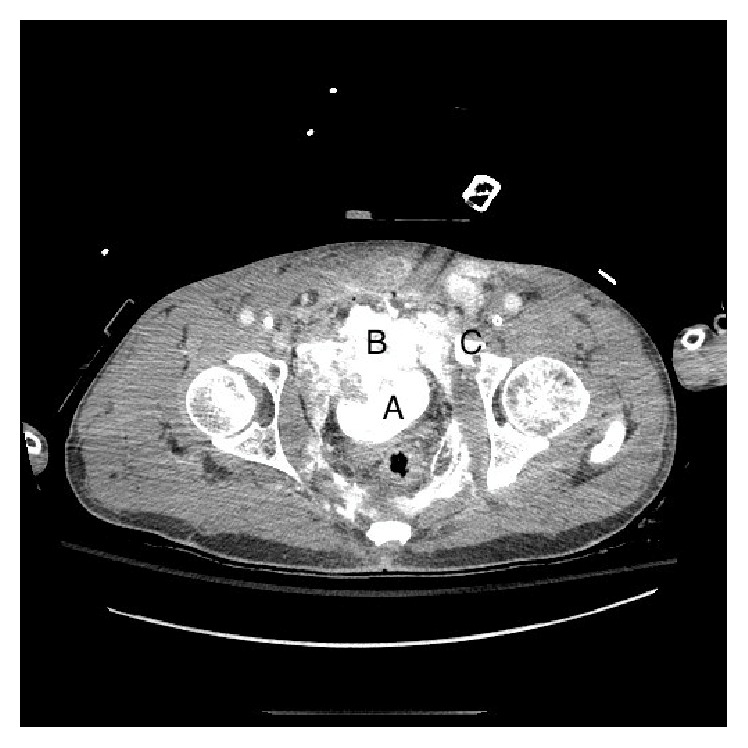
CT A/P demonstrating significant injuries in the pelvis including bladder injury with contrast extravasation and open book pelvic fractures. A: bladder filled with contrast. B: contrast extravasation anteriorly. C: fracture of the pubic ramus.

A 50-year-old man was brought in by ambulance after being run over by a semitruck. Upon initial evaluation, he was noted to have a pelvic crush injury with blood per urethral meatus. An attempt at urethral catheter placement was unsuccessful. A retrograde urethrogram demonstrated significant extravasation at the bladder neck, concerning for urethral disruption. A CT showed additional pelvic fractures and an open book fracture, multiple rectal injuries, and a small bowel injury with gross spillage of bowel contents into the abdomen. A laceration of the anterior bladder neck and extravasation of contrast into the pelvis was noted (Figure 4).

**Figure 5:**
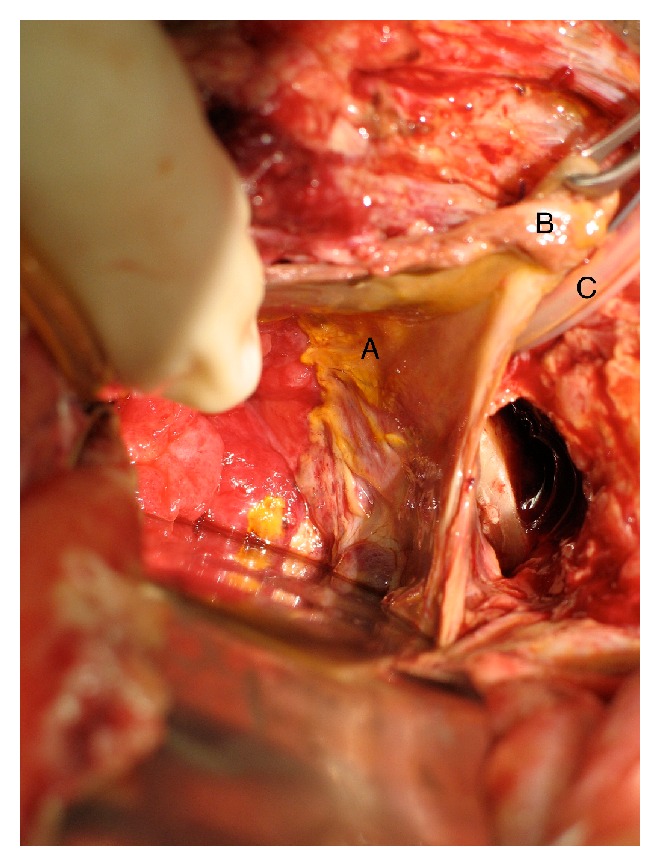
Key: A: necrotic tissue. B: bladder mucosa. C: Foley catheter.

He was taken emergently to the operating room for exploratory laparotomy. The areas of bowel injury were resected to healthy appearing tissue and the patient's bowels were left in discontinuity. He continued to have persistent hemorrhaging; therefore the abdomen was packed and he was taken to IR who embolized the bilateral internal iliac arteries. A suprapubic tube was placed in IR. Once stabilized, he was taken to the ICU. Forty-eight hours later, he was taken back to the operating room for reexploration. The suprapubic tube was noted to be in place and a flexible cystoscope was passed through the urethra. The urethra was noted to be intact to the level of the bladder neck and a council tip catheter placed over a wire. Exploratory cystotomy revealed a 3 cm bladder neck laceration extending from the anterior to right aspect of the bladder. No other injuries were identified. The surrounding tissue was normal (Figure 5). Multiple patchy areas dispersed throughout the bladder appeared gray and devitalized. Given the extent of the necrosis, we elected to not debride the unhealthy tissue and repaired the anterior bladder neck injury. A suprapubic tube was replaced and the bladder was closed in two layers. Creation of an end sigmoid colostomy with segmental sigmoid resection and complex abdominal wall closure was performed. IR placed bilateral nephrostomy tubes. He was stabilized and eventually discharged to a skilled nursing facility for recovery and rehabilitation. To date, he is doing well with a functional bladder and all tubes were subsequently removed.

## 4. Discussion

Bladder necrosis is one of many potential, albeit rare, sequelae of pelvic artery embolization. Pelvic arterial embolization has been utilized for various clinical scenarios including emergent management of significant bleeding in trauma patients with significant pelvic fractures and uncontrolled bleeding after gynecologic procedures or radiotherapy for management of pelvic tumors [4]. The bladder vascular supply derives from the anterior trunk of the internal iliac artery, which gives way to the superior and inferior vesical arteries. Prior literature has discussed known sequelae and has identified injuries to the bladder resulting in necrosis of bladder tissue due to decreased perfusion from these vessels [2, 4, 5, 7, 8]. A retrospective study by Matityahu et al. examined complications after pelvic angiographic embolization. The authors reported a complication rate of 11% after embolization, including one case of bladder necrosis out of 98 patients. All patients who experienced complications had undergone bilateral embolization. The authors discuss how the extent of embolization of the internal iliac arteries, bilateral compared to unilateral, may increase the risk of complications. In our reporting, both patients underwent bilateral internal iliac artery embolization prior to bladder necrosis being identified intraoperatively.

Bladder necrosis has been previously described as a delayed complication of pelvic arterial embolization, typically occurring 4-5 weeks after the procedure [2, 6]. Our reported cases differ in the acuity of this finding, which presented as early as two weeks after the initial trauma. There are many potential etiologies for this development including the initial traumatic injury, hypoxemia, hypovolemia, tissue hypoperfusion, and potential migration of embolization agents causing infarction which are likely contributing factors. The resulting vascular compromise and eventual bladder necrosis are likely multifactorial. Persistent hypoxia as a result of the embolization plus prolonged hypotension, inadequate collateral vascularization, and hypovolemia from the initial injuries may have led to compromised tissue viability. In our two cases, the rapid necrosis was a likely result of the significant initial vascular injuries combined with embolization.

In both cases presented, the initial bladder injuries are classified as Grades IV-V bladder injuries, requiring intraoperative repair and drainage via catheterization [9]. These cases highlight the variable degree of necrosis associated with these injuries with management including urinary diversion from the injured area with suprapubic catheterization. In many cases, nephrostomy tubes are utilized to preserve renal function and allow adequate drainage of the urinary tract as well as diverting antegrade drainage from the bladder. A simple cystectomy was required in one case given the extent of bladder injury and necrosis.

## 5. Conclusion

Bilateral internal iliac artery embolization may lead to bladder necrosis; however, this life-saving procedure is necessary following catastrophic pelvic trauma. These cases highlight the need to monitor the urinary tract following embolization and counsel patients on the associated risks. Management following postembolization bladder necrosis is dependent on patient stability and degree of necrosis.

## Abbreviations

CT: Computed tomography
CT A/P: Computed tomography abdomen/pelvis
ICU: Intensive Care Unit
IR: Interventional Radiology.
